# Endovascular Embolization of Pulmonary Sequestration in Children with Contraindications to Surgery: A Two-Centre Experience with Long-Term Follow-Up

**DOI:** 10.3390/children13060842

**Published:** 2026-06-22

**Authors:** Marcin Losin, Maciej Chojnicki, Weronika Lotkowska, Ewelina Wojciechowska, Maciej Murawski, Bartosz Regent, Piotr Czauderna

**Affiliations:** 1 Department of Surgery and Urology for Children and Adolescents, Medical University of Gdańsk, ul. Smoluchowskiego 17, 80-214 Gdańsk, Poland; 2 Laboratory of Pediatric Interventional Cardiology, St. Adalbert Hospital, COPERNICUS, 80-803 Gdańsk, Poland; 3 Department of Radiology, Medical University of Gdańsk, 80-210 Gdańsk, Poland

**Keywords:** pulmonary sequestration, endovascular embolization, pediatric interventional radiology, neonate, congenital lung malformation

## Abstract

**Highlights:**

**What are the main findings?**

- Endovascular embolization was technically successful in all six pediatric patients with pulmonary sequestration and contraindications to surgery, including a neonate treated at 11 days of life for high-output cardiac failure and a 6-week-old infant with Trisomy 21 and pulmonary hypertension. There were no major procedural complications.
- Embolization was followed by rapid and durable hemodynamic recovery as follows: reverse cardiac remodelling within five weeks in the patient with pre-procedural left ventricular dilation (Z-score +2.45 → normal), reduction in systolic pulmonary artery pressure from 35 to 40 mmHg to 17 mmHg within six weeks in the neonate, and normalization of the echocardiographic PAH signature by post-procedural day six in the infant treated under sildenafil.

**What is the implication of the main finding?**

- Endovascular embolization can be performed safely in very young infants and neonates with pulmonary sequestration when surgery is contraindicated, even in the presence of comorbidity such as pulmonary hypertension, congenital pneumonia or neonatal venous thrombosis on therapeutic anticoagulation.
- Documented reverse cardiac remodelling and rapid hemodynamic normalization suggest that, in selected pediatric cases, embolization alone may achieve durable clinical benefit without staged surgical resection. The indications for routine resection after EE deserve prospective re-evaluation.

**Abstract:**

**Background and Objectives:** Pulmonary sequestration (PS) is a rare congenital lung anomaly with anomalous systemic arterial supply. Surgical resection is the standard treatment, but some children have contraindications. Endovascular embolization (EE) is an established alternative; published pediatric experience is limited, particularly in neonates. We report a two-centre experience with extended follow-up and quantitative hemodynamic data. **Methods:** Six pediatric patients (five male; median age 6 months, range 11 days to 4 years and 8 months) underwent EE for PS at two centres in Gdańsk, Poland, between 2020 and 2025. Contraindications to surgery were severe pulmonary arterial hypertension, high-output cardiac failure, low body weight with comorbidity, complex extralobar anatomy or refused parental consent. Procedures were performed under general anesthesia via right common femoral arterial access; device strategy was tailored to vessel anatomy. **Results:** Technical success was 100% with no procedural complications. Median feeding-artery diameter was 3.4 mm (range 2.1 to 5.3 mm). An Amplatzer-family vascular plug was used in five patients (83.3%), pushable platinum coils in two (33.3%) and Onyx-18 in one (16.7%); two had hybrid combinations and one underwent planned staged two-step embolization. Median procedural duration was 51 min. At median follow-up of 50 months (range 11 to 68), all patients showed sequester regression on imaging. Reverse cardiac remodelling occurred within five weeks in the patient with pre-procedural left ventricular dilation (Z-score +2.45 returning to normal); systolic pulmonary artery pressure fell from 35 to 40 to 17 mmHg within six weeks in the neonate treated at 11 days of life for high-output cardiac failure. No patient required surgical resection. **Conclusions:** Endovascular embolization is safe and effective in pediatric patients with pulmonary sequestration and contraindications to surgery, including neonates with comorbidity. Documented reverse cardiac remodelling and rapid hemodynamic improvement support its use in selected cases.

## 1. Introduction

Pulmonary sequestration (PS) is an uncommon congenital malformation in which a portion of non-functioning lung tissue has no communication with the bronchial tree and is supplied by an aberrant systemic artery [1,2,3]. Reported incidence is 0.15% to 1.8% [1], and most cases are recognized in infancy. Two forms are distinguished by their relation to the visceral pleura and their venous drainage. Intralobar PS is the more frequent form, typically sitting in a lower lobe and draining into the pulmonary veins. Extralobar PS has its own pleural envelope and drains into systemic veins [1,2,3].

Prenatal ultrasound detects most cases today, and symptomatic neonates may require early intervention [1,3,4]. Surgical resection was for decades the default treatment [5,6]. Endovascular embolization (EE) has gained ground as a less invasive option, especially when the main goal is to close a left-to-right shunt or when operative risk is high [6,7,8,9]. Both techniques can be safe and effective, and the choice is usually driven by clinical presentation and associated lesions [1,6,7,8,9].

Pediatric series have reported good short-term outcomes after EE, including neonates, with resolution of shunt flow [8,9]. Data beyond two years remain thin. Most series come from single centres and use variable selection criteria and follow-up schedules. Guidance on when to choose EE over surgery in children, and in particular how to handle intralobar versus extralobar forms, is not yet standardized.

We report here our two-centre experience in children with contraindications to surgery, with a median follow-up of 50 months. The following four features distinguish this cohort from previously published pediatric EE series: a two-centre design, an extended median follow-up, routine use of hybrid device combinations, and patients at the youngest end of the spectrum—a neonate embolised at 11 days for high-output cardiac failure and a 6-week-old infant with Trisomy 21 and pulmonary hypertension on sildenafil. We describe how we select embolization devices and tailor treatment to lesion type.

## 2. Materials and Methods

### 2.1. Patient Population and Study Design

Six pediatric patients were treated between 2020 and 2025 at two tertiary centres in Gdańsk, Poland—five at the Department of Pediatric Cardiac Surgery, Copernicus Hospital, and one at the Department of Radiology, University Clinical Centre. The study was a retrospective, two-centre observational analysis. Five patients were male (83.3%) and one female (16.7%).

Only patients with contraindications to surgery were qualified for endovascular treatment. Contraindications to surgery in this cohort were severe pulmonary arterial hypertension in two patients, high-output cardiac failure in one neonate, very low body weight combined with significant comorbidity in three patients (all weighing ≤5.5 kg, including one with Trisomy 21 and one with a hybrid PS–CPAM lesion), complex extralobar anatomy with a subdiaphragmatic feeding vessel from the celiac trunk and concomitant Bochdalek hernia in one patient, and absence of parental consent for surgery in selected cases. Each treatment decision was made by the multidisciplinary team.

### 2.2. Lesion Characteristics and Associated Anomalies

Intralobar sequestration (ILS) was identified in four patients (66.7%) and extralobar sequestration (ELS) in two (33.3%). The sequestrum was most often located in the lower lobe of the left lung (two patients, both intralobar) or in the lower lobe of the right lung (two patients, both intralobar). One patient had a left subdiaphragmatic extralobar sequestration. One had an intrathoracic extralobar sequestration in the left hemithorax.

Associated anomalies and comorbidities included pulmonary arterial hypertension in two patients (33.3%), Trisomy 21 in one (16.7%), a hybrid PS–CPAM type II lesion in one (16.7%), horseshoe kidney with Bochdalek hernia and early-onset arterial hypertension in one (16.7%), and high-output heart failure as the indication for emergency embolization in one neonate (16.7%). Two patients (33.3%) had no significant associated anomaly at baseline.

### 2.3. Pre-Procedural Evaluation

Pre-procedural echocardiography showed tricuspid regurgitation in two patients as follows: trace TR with sPAP 25 mmHg in one, and moderate TR with sPAP 35–40 mmHg in the neonate with high-output cardiac failure and a dilated right ventricle. Another patient had a small left-to-right shunt at the foramen ovale and otherwise normal cardiac anatomy. One infant showed generalized cardiac enlargement with a left ventricular end-diastolic dimension of 28.2 mm (Z-score +2.45) and accelerated pulmonary vein inflow at 1.7 m/s, consistent with significant volume overload. The patient with Trisomy 21 had an abnormal pulmonary artery flow spectrum with shortened acceleration time (38 to 54 ms at heart rate 120/min), reflecting pulmonary hypertension treated with sildenafil. One patient had no significant pre-procedural echocardiographic abnormality.

All patients underwent computed tomographic angiography before the procedure to define arterial supply and venous drainage. Magnetic resonance angiography was added when vessel origin or course needed clarification, particularly for subdiaphragmatic feeding arteries. Pre-procedural echocardiography was performed in all cases. Ultrasonography was the main modality for post-procedural follow-up to limit radiation and sedation [4].

### 2.4. Endovascular Embolization Technique

All procedures were performed under general anesthesia in a dedicated pediatric interventional radiology suite. Vascular access was obtained through the right common femoral artery in all six patients. In one patient, additional right common femoral venous access was placed for invasive hemodynamic monitoring with a Swan–Ganz catheter (Edwards Lifesciences, Irvine, CA, USA—please confirm manufacturer/model), given pre-existing pulmonary hypertension on sildenafil. In the neonate, femoral arterial puncture was performed under ultrasound guidance because of low body weight (4.4 kg). Sheaths and catheters were 4F throughout, to match pediatric arterial diameters and to keep access-related trauma to a minimum. After sheath placement, heparin was given as a single intravenous bolus of 50 IU/kg. We used iodinated non-ionic contrast medium and capped cumulative dose at 4 mL/kg per procedure.

Diagnostic angiography was performed first, to define the origin, diameter, course and venous drainage of the aberrant feeding vessel. The feeding artery was then catheterised selectively, and the embolization strategy was tailored to vessel anatomy and operator judgement.

Three classes of embolic device were available. The Amplatzer Vascular Plug II or IV (Abbott Laboratories, Abbott Park, IL, USA) was the first-line device when feeding vessel diameter and proximal landing zone were favourable. Pushable platinum coils (IMWCE-3-PDA-3 and IMWCE-5-PDA-3; Cook Medical, Bloomington, IN, USA) were used in smaller or branching vessels. Onyx-18, an ethylene vinyl alcohol copolymer (Medtronic, Minneapolis, MN, USA), was reserved for cases with complex distal vascular architecture not amenable to mechanical occlusion alone. Specifically, Onyx-18 was preferred over a coil-based combination when the feeding vessel was small (approximately 2 mm or below) and divided into multiple small efferent branches as follows: in this anatomy coil deployment carries a real risk of incomplete occlusion through distal collaterals and of migration of pushable coils whose minimum diameter approaches the vessel calibre. The liquid embolic conforms to nidus-like distal architecture in a single deployment, at the cost of higher material cost and longer fluoroscopy time. Where intraprocedural angiography showed incomplete occlusion after a single device, we combined two or more devices in the same session.

Procedural duration was recorded from vascular access to sheath removal. The angiographic system tracked fluoroscopy time and air kerma–area product. Pediatric-specific low-dose protocols were used in all cases, with reduced frame rate (7.5 frames/s) and last-image-hold.

Endovascular embolization was performed selectively. The decision to offer EE rather than surgery was made by the multidisciplinary team after review of imaging and comorbidity profile and was applied only to patients with formal contraindications to resection or surgical risk considered prohibitive. EE was not used as routine first-line treatment for pulmonary sequestration at either institution.

### 2.5. Post-Procedural Management and Follow-Up

Post-procedural management included clinical monitoring and scheduled imaging follow-up with ultrasonography and echocardiography. Assessments were performed at 1, 3, 6 and 12 months, and yearly thereafter. In patients with suspected hybrid lesions (PS combined with congenital pulmonary airway malformation, CPAM), extended follow-up with repeated ultrasonography and chest CT was planned in accordance with current recommendations [3,10].

## 3. Results

### 3.1. Procedural Outcomes

All embolization procedures were technically successful. Median feeding-artery diameter was 3.4 mm (range 2.1 to 5.3 mm); in one patient a wide draining vein of 6.2 mm connected the sequester directly to the left atrium. Arterial supply arose from the descending thoracic aorta in two patients (33.3%), from the abdominal aorta with subdiaphragmatic origin in three (50%) and from the left gastric artery as a branch of the celiac trunk in one (16.7%). The three subdiaphragmatic feeding vessels all crossed the diaphragm to supply intrathoracic sequester tissue. Venous drainage was into the pulmonary veins in two patients (33.3%), directly into the left atrium through anomalous connections in three (50%), including the 6.2 mm draining vein, and into the gastric venous system in one (16.7%). Patient and procedural characteristics are summarized in Table 1.

Device selection adhered to the following framework described in Section 2.4: vessel calibre, take-off geometry and number of distal branches drove the choice between a vascular plug, coils and liquid embolic. An Amplatzer-family vascular plug was used in five patients (83.3%). Pushable platinum coils were used in two patients (33.3%) and Onyx-18 liquid embolic in one (16.7%). Hybrid embolization combining coils and a vascular plug in the same session was performed in two patients (33.3%). One patient underwent planned two-step staged embolization, with the second procedure performed about eight months after the first, using the same device class. In two further patients, selective closure of distal vessel divisions required deployment of multiple vascular-plug devices in a single session (three and two devices, respectively) [8,9].

Median procedural duration was 51 min (range 35 to 100 min). No intraoperative complications occurred. There was no access-site complication, no device-related adverse event and no immediate post-procedural hemodynamic instability.

### 3.2. Periprocedural Course

No patient developed post-embolization syndrome such as transient fever, pain or inflammatory symptoms. Device migration was not observed during early or long-term follow-up, despite ongoing somatic growth.

### 3.3. Long-Term Outcomes

Median follow-up was 50 months (range 11 to 68 months). One patient had short-term follow-up only because the procedure was performed recently. No patient experienced recurrent pulmonary infection, haemoptysis or respiratory distress. Serial imaging showed complete involution or significant regression of the sequestered lung tissue in every patient. No residual abnormal vascularisation was seen.

One patient underwent staged two-step embolization. Echocardiography at approximately seven months after the initial procedure showed persistent left ventricular enlargement, and ultrasonography demonstrated residual flow through the feeding vessel, attributed to collateral recruitment. A repeat embolization was performed at about eight months from the initial procedure, using the same device class. Left ventricular dimensions normalized at six and seventeen months after the second embolization, and complete vascular occlusion was confirmed on serial imaging.

**Table 1:** Patient and procedural characteristics of the six pediatric patients treated with endovascular embolization for pulmonary sequestration.

Pt	Age at EE	Sex	Type	Location	Feeding Artery	Venous Drainage	Associated Anomalies/Comorbidities	Device(s)	Dur. (min)	Vessel Ø (mm)	FU Outcome
1	4 y 8 mo	M	ELS	Subdiaphragmatic, left	Left gastric artery (branch of celiac trunk)	Gastric veins	Horseshoe kidney; Bochdalek hernia; early-onset arterial hypertension	Onyx-18 (single session)	38	~2.1	Embolization recent (3 June 2025) at second institution; sequester regression on serial US to 9 mo post-EE
2	8 mo	M	ILS	Lower lobe, left lung	Thoracic aorta	Pulmonary veins	None significant (pre-EE history of aspiration and obstructive bronchitis)	AVP × 2 (staged 2-step: first session + repeat at ~8 mo for persistent LV enlargement on FU echo)	35 (1st)	5.3	Persistent LV enlargement after 1st EE → repeat EE → LV dimensions normalized at +6 and +17 mo; trace TR
3	6 wk	F	ILS	Lower lobe, right lung	Thoracic aorta (at diaphragm level)	Pulmonary veins	Trisomy 21; severe pre-EE PPHN (PV acceleration time 38–54 ms; on sildenafil); congenital pneumonia with *Staphylococcus haemolyticus* sepsis; pre-existing neonatal DVT from ECC catheter (left iliofemoral, treated with heparin, in regression at EE)	Coils (3× IMWCE: 3PDA3, 3PDA5, 5PDA3) + AVP	100	3.4	Resolution of PAH signature at +6 d post-EE (MPA Vmax 1.2 m/s); invasive pre-EE Ao 55/35/PA 15/12 → post-EE Ao 73/52/PA 30/19 mmHg; pre-existing DVT showed continued recanalization on Doppler under prophylactic anticoagulation
4	4 mo	M	ILS + CPAM type II (hybrid)	Lower lobe, left lung	Abdominal aorta (subdiaphragmatic)	Left atrium (direct anomalous drainage)	CPAM type II (hybrid lesion); patent ductus arteriosus; prenatally diagnosed cardiac enlargement; mother with paroxysmal AF on Isoptin pre-delivery	AVP × 3 (three Amplatzer occluders in single session)	50	2.8 (pre-EE)/ 3.5–4 (peri-EE)	Reverse cardiac remodelling: LVDd 28.2 mm (Z +2.45) pre-EE → normal dimensions at +5 wk → preserved size with somatic growth to LVDd 34.5 mm at 20 kg, +58 mo; PV inflow 1.7 m/s pre-EE → undetectable post-EE; vessel exclusion durable
5	9 mo	M	ILS	Lower lobe, right lung	Abdominal aorta (subdiaphragmatic)	Left atrium (direct)	None significant at baseline (PFO on echo, isolated; choroid plexus cyst 2.5 mm)	AVP × 2 (selective branch closure of two distal divisions)	52	3.6 (origin)/ 2.4 + 2.6 (branches)	Normal echo at +4 d, +17 mo, +29 mo and +60 mo; sequester regression on serial US; persistent right axis deviation on ECG across follow-up; soft systolic PV-area murmur 2/6 at +60 mo (clinically innocent)
6	11 days (neonate)	M	ELS	Intrathoracic, left	Abdominal aorta (subdiaphragmatic)	Left atrium (via wide draining vein, 6.2 mm)	High-output heart failure (primary indication); pre-EE PAH (sPAP 35–40 mmHg, moderate TR with thickened tricuspid valve leaflets, dilated RV); patent ductus arteriosus; mother with paroxysmal AF on Isoptin pre-delivery	Coils (2× IMWCE: 3PDA4, 5PDA4) + AVP	66	3.4 (arterial)/ 6.2 (venous)	Resolution of PAH at +6 wk post-EE (sPAP 35–40 → 17 mmHg); trace residual L→R shunt at foramen ovale; discharged on triple heart-failure therapy (lisinopril + spironolactone + bisoprolol)—clinical wean of therapy and long-term surveillance ongoing

AVP, Amplatzer Vascular Plug; ELS, extralobar sequestration; EE, endovascular embolization; F, female; FU, follow-up; ILS, intralobar sequestration; L, left; LLL, left lower lobe; M, male; mo, months; Pt, patient; RLL, right lower lobe; y, years.

Cardiac remodelling and pulmonary hypertension responded promptly to embolization in patients with documented baseline abnormalities. In the infant with pre-procedural LV dilation (Z-score +2.45) and accelerated pulmonary vein inflow at 1.7 m/s, echocardiographic dimensions returned to age-appropriate values within five weeks. The pulmonary vein inflow signal was no longer detectable post-procedure, and LV dimensions remained appropriate for somatic growth at 58-month follow-up. In the neonate embolised at 11 days of life for high-output cardiac failure, follow-up echocardiography at six weeks showed reduction in sPAP from 35 to 40 mmHg to 17 mmHg, and the patient was discharged on triple heart-failure therapy with subsequent clinical improvement. In the patient with pre-procedural pulmonary hypertension on sildenafil, the PAH signature on echo normalized by post-procedural day six (MPA Vmax 1.2 m/s). Invasive hemodynamic measurements during this procedure showed aortic pressure rising from 55/35 to 73/52 mmHg after device deployment, consistent with elimination of low-resistance systemic-to-pulmonary shunt flow. The patient who underwent staged two-step embolization had a pre-procedural history of aspiration and obstructive bronchitis; no comparable events were reported during long-term follow-up.

### 3.4. Representative Procedural Imaging

**Figure 1:**
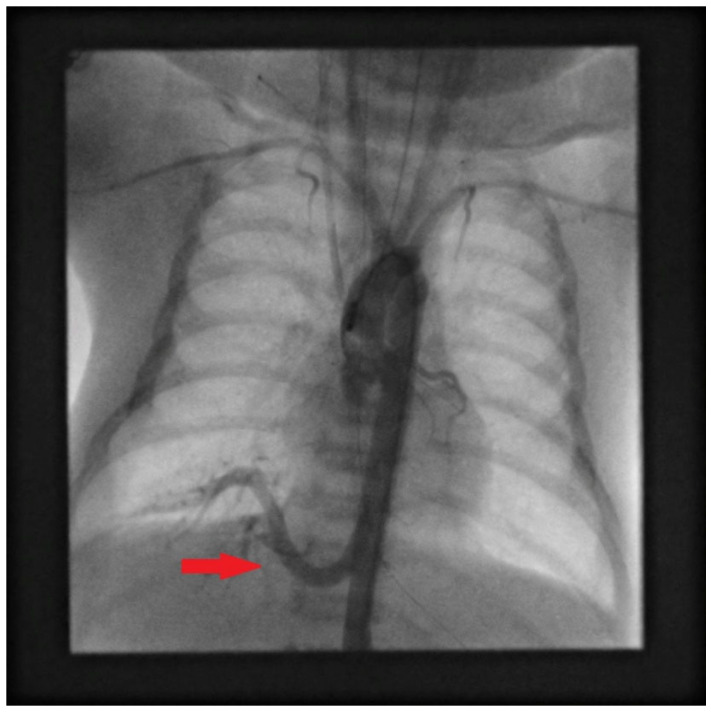
Baseline digital subtraction angiography in a 6-week-old infant with intralobar pulmonary sequestration of the right lower lobe, performed via right femoral arterial access. An aberrant systemic artery arises from the descending thoracic aorta and runs inferolaterally, with distal branching into the sequestered parenchyma. A central venous line and ECG leads are also visible. The red arrow indicates the aberrant systemic feeding artery arising from the descending thoracic aorta.

**Figure 2:**
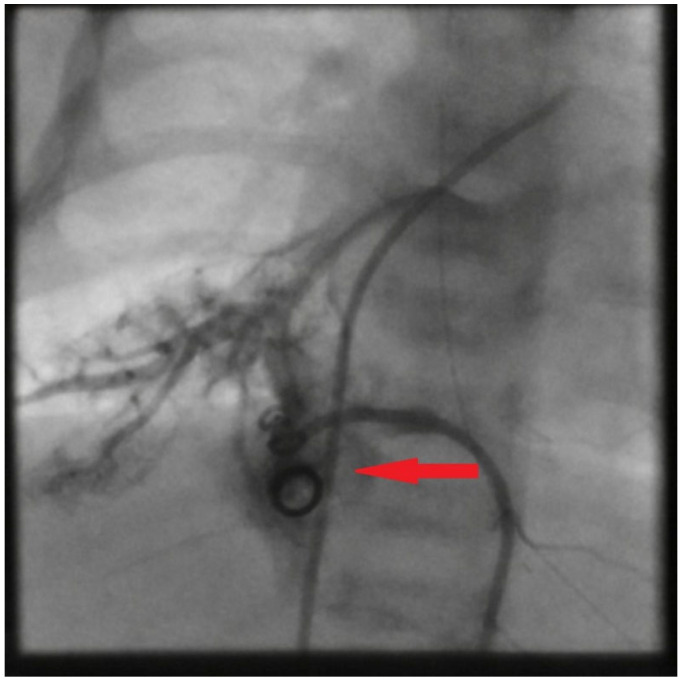
Selective angiography after initial coil deployment. The coils are visible in the proximal-mid segment of the aberrant feeding artery. Small distal branches still opacify, which shows that occlusion is incomplete and that an additional device is needed. This image captures the intermediate stage of the hybrid approach, before placement of the Amplatzer Vascular Plug. The red arrow indicates the residual opacification of the small distal branches, confirming incomplete occlusion before placement of the Amplatzer Vascular Plug.

**Figure 3:**
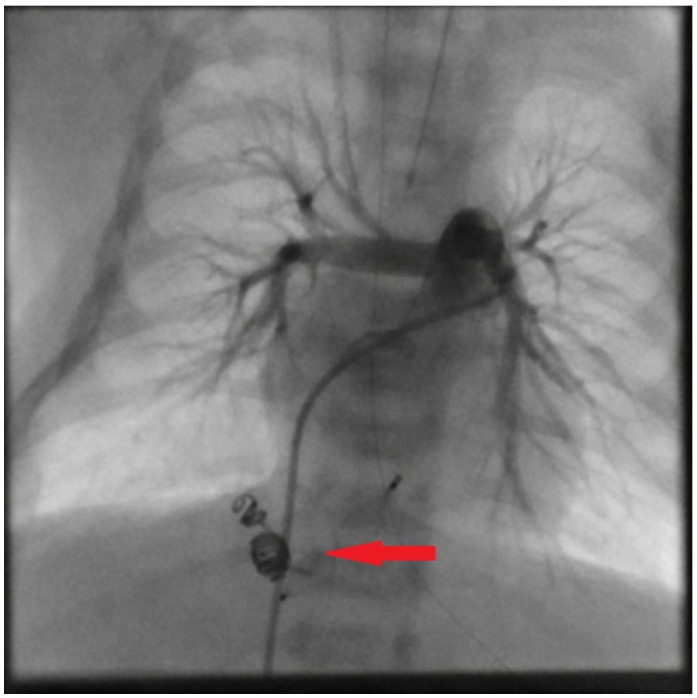
Final DSA after hybrid embolization (coils plus Amplatzer Vascular Plug). The aberrant feeding vessel is completely occluded. Bilateral pulmonary perfusion is preserved, and the pulmonary arterial tree fills normally. No contrast reaches the sequestered parenchyma. The red arrow indicates the site of the now completely occluded aberrant feeding vessel.

Procedural angiograms from the embolization of an intralobar pulmonary sequestration in a 6-week-old infant (Patient 3) are shown in Figure 1, Figure 2 and Figure 3.

## 4. Discussion

Management of pulmonary sequestration in children is not straightforward: decisions depend on lesion type, patient age and vascular anatomy. Our experience, read alongside the literature, fits the broader move away from open surgery toward minimally invasive options and, in some cases, combined interventional-surgical approaches [1,5,6,7,8,9].

### 4.1. Treatment Considerations by Lesion Type

ELS and ILS call for different therapeutic considerations [1,2,3]. ELS has its own pleural envelope and is well demarcated, which makes limited resection feasible without sacrificing functioning parenchyma. Surgical outcomes for ELS are good, with low operative risk in experienced pediatric centres. ILS is a different surgical problem. Most ILS cases in children require lobectomy because segmental resection is technically demanding in small patients. Open thoracotomy was historically the standard, but contemporary pediatric thoracic surgery increasingly uses thoracoscopic lobectomy in selected centres, with shorter hospital stay and better cosmesis [1,11]. In experienced hands, surgery remains the most definitive option. It provides histopathological confirmation of the lesion, removes any risk of recanalisation, and has well-documented long-term safety. The main trade-off in ILS is loss of functioning parenchyma after lobectomy, plus the morbidity of open thoracotomy where minimally invasive surgery is not available [5,12].

Robotic-assisted thoracic surgery is an emerging option. Its use remains limited in very small infants because of working-space and instrument-size constraints [13]. Where available, thoracoscopy and robotic approaches narrow the surgical morbidity gap that has historically motivated interest in endovascular alternatives.

### 4.2. Rationale for Endovascular Embolization

The reason to intervene in PS at all is the aberrant systemic artery, which shunts blood from the systemic to the pulmonary circulation and can lead to high-output cardiac failure. Both surgery and EE address this. Interest in EE has grown over the past decade [8,9] for cases where surgery is contraindicated, where operative risk is judged prohibitive, or where EE is used as a bridge to elective resection.

Our cohort had 100% technical success and the associated symptoms resolved during follow-up. These results match previously published pediatric series, including ones with neonates and infants with multiple comorbidities [1,8,9]. EE avoids the parenchymal loss inherent to lobectomy and the chest-wall morbidity of open thoracotomy. These can matter especially in ILS, in premature infants, and in patients with bilateral disease [8,9]. EE has its own downsides, namely risk of recanalisation, no histopathological tissue, and need for prolonged imaging follow-up; we address these in Section 4.5.

### 4.3. Comparison with the Previous Literature

Several pediatric series have reported EE outcomes for PS over the past ten years. Table 2 compares them with our data.

Our series is smaller than Abu Zahira’s [9]. It differs in four respects. It comes from two independent institutions, not one. The follow-up is longer, with a median of 50 months against roughly 24 months in the French cohort. We used a wider range of devices, including Onyx-18 and hybrid combinations that earlier series did not include. Also, we restricted EE to children with contraindications to surgery; published cohorts often mix indications. Our single recurrence (1/6, 16.7%) was caused by collateral recruitment and not by device failure. Notably, this is well below the 25–47% pooled recurrence range quoted by Saxena et al. [9], which is why they explicitly recommend planned surgical resection after EE. The gap probably reflects our device choice (Amplatzer Vascular Plugs and hybrid combinations rather than coils alone) and our selection of smaller feeding vessels, but structured imaging follow-up is clearly not optional. Recurrence detection in any series of this size is sensitive to both follow-up duration and surveillance density as follows: a longer median follow-up increases the chance of catching late residual flow, while irregular surveillance intervals (see Section 4.6) may have masked transient or subclinical residual perfusion in patients with sparser sampling.

### 4.4. Clinical Message: When to Consider Endovascular Embolization

**Figure 4:**
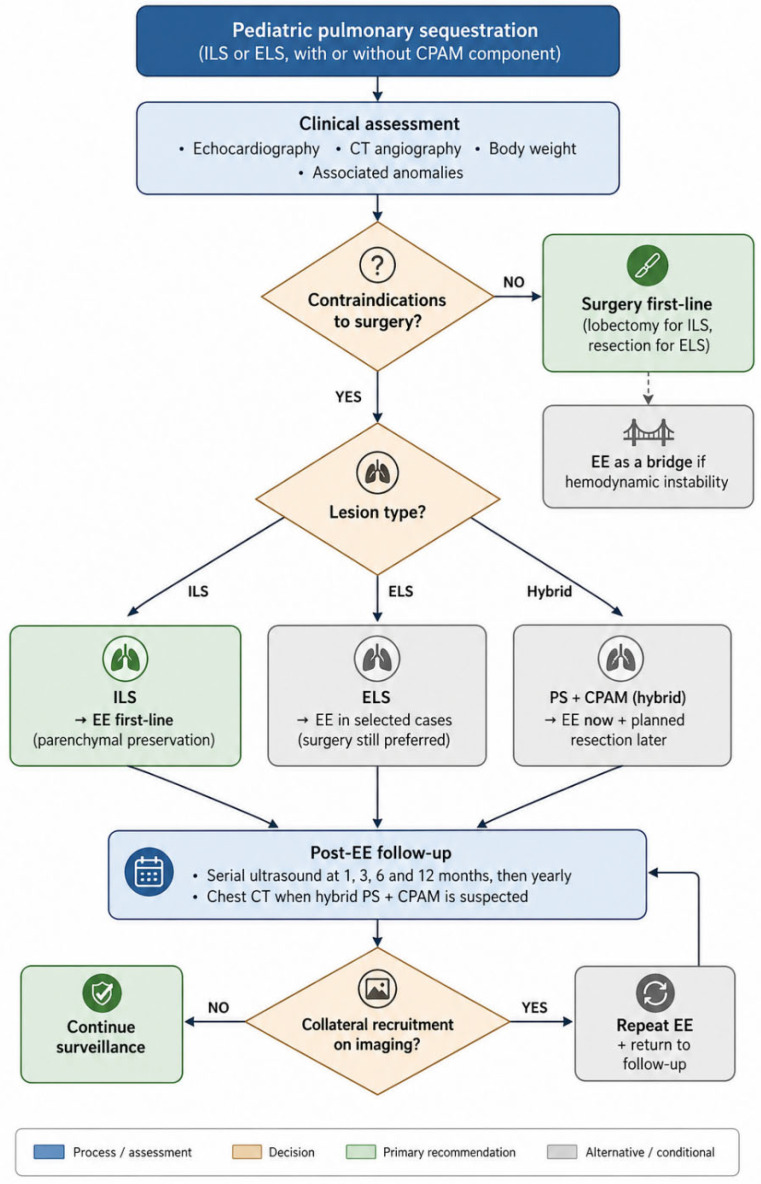
Proposed lesion-type-based framework for considering endovascular embolization (EE) in pediatric pulmonary sequestration. The diagram is intended to support multidisciplinary decision-making and is based on the authors’ two-centre experience together with the existing literature; it should not be interpreted as a standard of care. ILS, intralobar sequestration; ELS, extralobar sequestration; PS, pulmonary sequestration; CPAM, congenital pulmonary airway malformation.

From our experience and the existing literature, a lesion-type-based framework is reasonable. The framework is summarized in Figure 4 and is meant to support multidisciplinary discussion. It does not replace it, and it is offered as current evidence rather than as a standard of care. The caveats are in Section 4.6.

EE may be considered as the first intervention in ILS with a hemodynamically significant aberrant feeding artery when surgery is contraindicated. Other situations that may favour EE include thoracotomy with unacceptable perioperative risk (severe pulmonary hypertension, complex congenital heart disease) and very low-weight infants in whom resection is technically difficult. Lung-parenchymal preservation can also matter in bilateral disease and in preterm infants.

EE may also be used as a bridge before elective surgical resection. Pre-operative occlusion of the aberrant artery reduces intraoperative bleeding and can sometimes make a lung-sparing operation feasible.

Surgery is still the default in most extralobar lesions, where well-demarcated anatomy and absence of pleural-shared parenchyma favour resection at low operative risk. It is also preferred when PS is associated with CPAM, because histopathological tissue evaluation is then meaningful [3,10]. Anatomical features that predispose to recanalisation, such as a feeding vessel above 5 mm in diameter or multivessel arterial supply, also tip the balance back toward surgery [8,9].

Our cohort was preselected on contraindications to surgery. Our data therefore describe how safe and durable EE is in a high-risk pediatric subgroup. They do not show that embolization is equivalent or superior to surgery in the broader pediatric PS population.

### 4.5. Technical Considerations and Procedural Caveats

EE has its own downsides. It avoids thoracotomy-related chest-wall morbidity but brings a small risk of thrombosis and femoral artery stenosis, which can impair limb perfusion and, in extreme cases, lead to growth asymmetry of the lower limb. Long-term follow-up is therefore warranted [9]. In our series EE was safe even in neonates and in patients with pulmonary hypertension, as long as device selection matched vessel anatomy [8,9].

A few technical caveats apply. Feeding vessels over 5 mm are prone to recanalisation, and Saxena et al. [9] report pooled recurrence rates of 25–47% across published embolization series, which is why those authors advocate a two-step approach of EE.

Blood flow can return through previously occluded vessels. We saw this in one of our patients: collateral recruitment required a second embolization. Regular clinical and imaging follow-up is therefore essential. For routine surveillance, ultrasound is usually enough and has the added advantage of avoiding anesthesia. Where CPAM is suspected alongside PS, CT or MRI eventually become necessary, but in small children these examinations require sedation and are best postponed. Our protocol relies on serial ultrasound initially and reserves CT for later time points when clinically indicated.

Hybrid lesions (PS with CPAM) are a different problem. One patient in our cohort had a hybrid ILS + CPAM type II lesion. Embolization in this case achieved durable closure of the systemic feeding vessel with documented reverse cardiac remodelling; the CPAM component remains under serial imaging surveillance. Embolization alone does not suffice in hybrid lesions, because the CPAM component carries a malignancy risk and requires tissue diagnosis [3,10]. When EE is performed first to address the hemodynamic burden, surgical resection of the residual tissue should still be planned once the child is stable enough. Where the hybrid lesion is not surgically resected, our practice is cross-sectional imaging (CT or MR) at two- to three-year intervals through childhood, with a planned CT around the time of transition to adult care (approximately 18 years of age), and continued surveillance thereafter decided case by case in collaboration with adult pulmonology. We do not set a single age at which surveillance can be safely discontinued, because reports of pleuropulmonary blastoma in early childhood and of mucinous adenocarcinoma into adulthood make a single safe cutoff difficult to defend. Patients with hybrid PS–CPAM are not formally discharged from imaging follow-up.

Preoperative EE followed by thoracoscopic resection is an attractive hybrid option in complex cases [1,8,9]. It reduces intraoperative bleeding and can make lung-sparing resection feasible. There is the following trade-off: embolic material triggers local inflammation and changes tissue planes, which can make the subsequent operation technically harder [9].

### 4.6. Limitations

Several limitations of this work should be weighed when reading our findings.

This study should be read primarily as a case series rather than a comparative outcome study. Six patients is very small. With this number, point estimates for technical success and recurrence carry wide confidence intervals. Subgroup analysis (ILS vs. ELS, age strata, and device type) is not statistically meaningful. The favourable outcomes here cannot be taken as evidence of treatment efficacy in any statistical sense.

Our cohort is highly selected. Children were eligible for EE only if they had formal contraindications to surgery or if parents declined surgery. Most children with pulmonary sequestration treated at our centres during the same period went to surgical resection. The favourable results we report should therefore not be extrapolated to children for whom surgery is feasible.

We did not attempt a comparative analysis with surgical group from the same period and centres. Comparisons with published surgical or embolization series are limited by differences in patient selection, lesion characteristics, follow-up protocols, and reporting standards. Our suggestion that staged surgical resection after EE may not be necessary in all children is a hypothesis from uncontrolled retrospective data, not a recommendation backed by comparative evidence.

Retrospective design adds the following usual limits: incomplete data capture, follow-up protocols not standardized between the two centres, and clinician choice of imaging modality at each visit. We assessed outcomes through clinical review and imaging, neither of which catches occult complications or asymptomatic residual disease.

EE leaves no tissue for histopathology. Occult malignant transformation cannot be ruled out, particularly in patients with hybrid PS–CPAM lesions [3,10]. These children cannot be discharged from follow-up; structured, continuous imaging surveillance is mandatory, with responsibility for that surveillance assigned explicitly at the point of treatment. The data come from two centres in northern Poland. Local referral patterns, surgical preferences and population characteristics may shape our results. External validity to other healthcare systems is unknown.

A median follow-up of 50 months is longer than most published pediatric embolization series, but it is still too short to evaluate outcomes into adolescence and adulthood. Long-term effects of femoral arterial access in early infancy and any delayed effects on lung development would only become apparent later.

Respiratory symptoms were assessed by qualitative clinical review and parent-reported questionnaire at each follow-up visit, rather than by structured pre-/post-comparison of infection frequency, exercise tolerance or oxygen requirement. One patient had a pre-procedural history of aspiration and obstructive bronchitis that did not recur during follow-up. No patient reported recurrent pulmonary infection, haemoptysis or respiratory distress in the long term. Formal quantitative respiratory outcome data are, however, not available.

Our pre-specified follow-up schedule called for echocardiographic and ultrasonographic assessment at 1, 3, 6 and 12 months and yearly thereafter. In practice, the actual intervals between assessments were not uniform across patients, reflecting the retrospective design and routine clinical scheduling. Some patients had dense longitudinal data, with one patient assessed at twelve separate time points over 58 months, while others had sparser sampling. One patient was embolised recently and had short-term follow-up only. This heterogeneity limits direct comparison of the timing of cardiac remodelling and shunt-physiology resolution across patients.

One patient had pre-existing neonatal venous thrombosis (left iliofemoral, originating from an extracorporeal catheter placed during neonatal intensive care for severe respiratory failure) that was being managed under therapeutic anticoagulation at the time of embolization. The embolization was performed through right-sided femoral access and was uncomplicated; Doppler ultrasonography during the embolization hospitalization documented continued recanalization of the thrombosed venous segment. This experience suggests that EE can be performed safely in low-weight infants with concurrent thrombotic comorbidity under appropriate anticoagulation, but larger series will be needed to define the true risk profile in this population.

Larger prospective multicentre studies, with predefined follow-up protocols and ideally with parallel surgical cohorts, will be needed to refine the indications for EE in pediatric pulmonary sequestration and to test the hypotheses that our data raise.

## 5. Conclusions

In this two-centre retrospective case series of six children with pulmonary sequestration and contraindications to surgery, transcatheter endovascular embolization was performed without major complications. The cohort included a neonate treated at 11 days of life for high-output cardiac failure and a 6-week-old infant with Trisomy 21 and pulmonary hypertension. Imaging follow-up extended beyond 50 months in four patients, with regression or involution of the sequestrum in every case. One patient underwent staged two-step embolization indicated by persistent left ventricular enlargement on follow-up; subsequent imaging showed complete vascular occlusion. None of the six patients required surgical resection.

Our small, selected cohort suggests that EE can be a reasonable option in carefully chosen pediatric patients in whom surgery is not feasible. It does not address whether EE is comparable to surgery in children for whom both options are open. Only adequately powered comparative studies can answer that question.

Saxena and colleagues [9] have argued for routine surgical resection after EE on the basis of pooled recurrence rates of 25 to 47% in earlier series. We do not read our findings as evidence against that recommendation. They do, however, raise the following research question: with modern occlusion devices and structured ultrasound surveillance, can a subset of children be managed with EE and active follow-up alone, without staged resection? This is worth a prospective study.

Until that evidence exists, surgery should remain the default option for children with pulmonary sequestration who can safely undergo it, in particular for extralobar lesions and for cases with suspected hybrid PS–CPAM features. EE is worth further evaluation as an alternative for children with contraindications to surgery, and as a bridge to elective resection in hemodynamically unstable patients. Long-term imaging follow-up is essential in either case.

## Figures and Tables

**Table 2:** Comparison of selected pediatric series reporting outcomes of endovascular embolization for pulmonary sequestration.

Study	Year	Design/Setting	n	Age Range	Devices	Median FU	Tech. Success/Recurrence
Ríos-Méndez et al. [7]	2017	Single-centre, Ecuador	10	4 mo–12 y	Coils	~20 mo	100%/~10%
Zhang et al. [6]	2017	Single-centre, China (surgery vs. EE)	28 (subset EE)	Pediatric + adult	Coils, plugs	Variable	High/NR
Khen-Dunlop et al. [1]	2018	Single-centre, France (Necker, 2000–2015)	99 (46 EE, 59 surgery)	Pediatric	Coils (EE); surgery (23 open, 36 thoracoscopic)	NR	13% needed secondary resection after EE
Abu Zahira et al. [8]	2023	Single-centre, France	13	Neonate–adolescent	Coils, plugs	~24 mo	100%/~8%
Saxena et al. [9]	2023	Letter to Editor (Radiographics)	N/A (no own cohort)	N/A	PVA, coils, AVP, gel foam	N/A	Reports recurrence 25–47% (the pooled literature)
Present study	2026	Two-centre, Poland	6	11 d–4 y 8 mo	AVP, coils, Onyx-18, hybrid	50 mo	100%/16.7%

AVP, Amplatzer Vascular Plug; EE, endovascular embolization; FU, follow-up; mo, months; N/A, not applicable; NR, not reported; y, years.

## Data Availability

The data presented in this study are available on reasonable request from the corresponding author. The data are not publicly available due to privacy and ethical restrictions.
